# Supplementary material for: Analysis of PPARγ Signaling Activity in Psoriasis
Source: Int J Mol Sci. 2021 Aug 10;22(16):8603. doi: 10.3390/ijms22168603 (PMC8395241; doi:10.3390/ijms22168603)
Supplement: Supplementary file 1 [file ijms-22-08603-s001.zip › Supplemental materials_Analysis of PPARg signaling activity in psoriasis/Pathway models/Models images and html files/PPARG expressed in cells assotiated with psoriasis/115946.html]

vascular endothelium


# Tissue vascular endothelium

|  |  |
| --- | --- |
| URN | urn:agi-ncimtissue:C0014261 |
| Total Entities | 3 |
| Connectivity | 2834 |
| Name | vascular endothelium |

---

|  |  |
| --- | --- |
| ChildConcepts | glomerular capillary endothelium |
|  | blood vessel endothelium |
|  | basal lamina of the vascular endothelium |

---

|  |  |
| --- | --- |
| Pathway | PPARG expressed in cells assotiated with psoriasis |

---

|  |  |
| --- | --- |
| MedScan ID | 6000107 |

---

|  |  |
| --- | --- |
| Alias | vascular endotheliums |
|  | vascular endothelium |

---

|  |  |
| --- | --- |
| NCIm ID | C0014261 |

---
